# *Ex Vivo* Modeling of Human Neuroendocrine Tumors in Tissue Surrogates

**DOI:** 10.3389/fendo.2021.710009

**Published:** 2021-12-23

**Authors:** Brendon Herring, Samuel Jang, Jason Whitt, Kayla Goliwas, Zviadi Aburjania, Vikas Dudeja, Bin Ren, Joel Berry, James Bibb, Andra Frost, Herbert Chen, John Bart Rose, Renata Jaskula-Sztul

**Affiliations:** ^1^ Department of Surgery, University of Alabama at Birmingham, Birmingham, AL, United States; ^2^ Department of Medicine, University of Alabama at Birmingham, Birmingham, AL, United States; ^3^ Department of Biomedical Engineering, University of Alabama at Birmingham, Birmingham, AL, United States; ^4^ Department of Pathology, University of Alabama at Birmingham, Birmingham, AL, United States

**Keywords:** neuroendocrine tumor, bioreactor 3D culture, biomedical engineering (BME), tumor surrogates, tumor modeling

## Abstract

Few models exist for studying neuroendocrine tumors (NETs), and there are mounting concerns that the currently available array of cell lines is not representative of NET biology. The lack of stable patient-derived NET xenograft models further limits the scientific community’s ability to make conclusions about NETs and their response to therapy in patients. To address these limitations, we propose the use of an ex vivo 3D flow-perfusion bioreactor system for culturing and studying patient-derived NET surrogates. Herein, we demonstrate the utility of the bioreactor system for culturing NET surrogates and provide methods for evaluating the efficacy of therapeutic agents on human NET cell line xenograft constructs and patient-derived NET surrogates. We also demonstrate that patient-derived NET tissues can be propagated using the bioreactor system and investigate the near-infrared (NIR) dye IR-783 for its use in monitoring their status within the bioreactor. The results indicate that the bioreactor system and similar 3D culture models may be valuable tools for culturing patient-derived NETs and monitoring their response to therapy *ex vivo*.

## 1 Introduction

Neuroendocrine tumors (NETs) are heterogeneous neoplasms originating in diverse anatomical locations, including the lungs, liver, intestines, and adrenal glands. NETs constitute over 2% of malignancies, and their incidence and prevalence is steadily increasing (1). For example, gastroenteropancreatic NETs (GEP-NETs) recently became the second most prevalent gastrointestinal (GI) malignancy after colorectal cancer (2, 3). Their variable presentations and relative rarity, at a yearly incidence of 6.8 cases per 100,000 people, can complicate diagnosis (2). Aberrant bioactive substance secretion by NETs can cause debilitating symptoms such as diarrhea, flushing, cardiac valvular lesions, and metabolic abnormalities (4). However, most NETs are asymptomatic prior to the development of metastases, which are present at diagnosis in 58% of patients (5). Widespread metastasis at presentation often make complete resections impossible, necessitating systemic therapy (6).

Effective systemic therapies for NETs remain scant, as the slow growth of well-differentiated NETs in particular limits the efficacy of cytotoxic chemotherapy (7). Additionally, the few systemic therapies proven clinically useful, including somatostatin analogues, everolimus, sunitinib, and Peptide Receptor Radionuclide Therapy (PRRT), can have widely variable response rates and debilitating side effects (8–13). Development of effective systemic therapies is hampered by the deficiency in tools for studying NETs. Few publicly available human-derived NET cell lines exist, and establishing patient-derived xenografts (PDX) has proven difficult, yielding success rates as low as 1/106 in a recent study (14, 15). This lack of models has similarly affected the ability of researchers to study various unsolved aspects of NET biology. Among these are the role of the complex tumor immune microenvironment and the effects of various genomic alterations on the behavior and progression of NETs (16–19).

**Figure 1:**
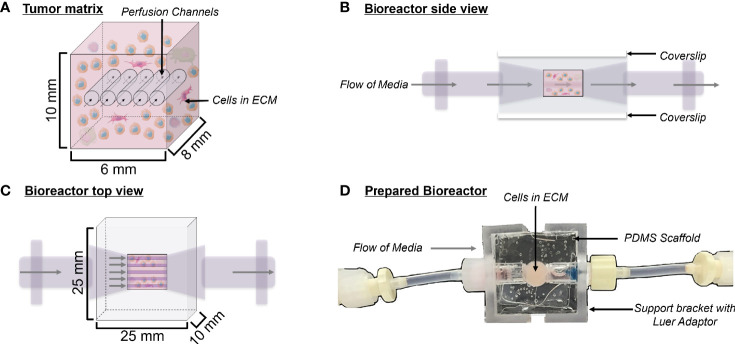
Bioreactor design. **(A)** Schematic depicting a cross-section of the tumor matrix volume, containing cells within an extracellular matrix (light pink) pierced by five perfusion channels. Flow of media through these channels is indicated by arrows. The composition of the surrogate ECM includes Matrigel, collagen (type 1 bovine), DMEM, sodium bicarbonate, cancer cells, and stroma. **(B)** Side view schematic of the bioreactor with cells in culture, showing coverslips on the top and bottom surfaces, which allow for non-invasive imaging during culture. **(C)** Top view schematic of the bioreactor with cells in culture. **(D)** Top view photograph of the polydimethylsiloxane bioreactor final product. Support brackets on each side attach to Luer fittings and hold guide wires leading to the perfusion channels within the tumor matrix.

To overcome these limitations and the lack of tissue-similar NET models, we propose the employment of a 3D flow-perfusion bioreactor system (**Figure 1**), previously characterized as a model system of breast and lung cancer, as an *ex vivo* model for studying NET biology and therapeutics (20, 21). The model consists of a 3D polydimethylsiloxane (PDMS) scaffold housing a homogenous mixture of tumor cells and extracellular matrix (ECM), which is constantly perfused with nutrient media. Herein, patient-derived bioreactor specimens are termed tumor *surrogates*, while cell-line/xenograft derived specimens are termed tumor *constructs*. The design considered the observations that mechanical and paracrine interactions with adjacent cells, ECM properties, and 3D structure can influence the phenotype and therapeutic response of cells in culture (22–25). These interactions may explain why many therapeutics with promising efficacies in preclinical 2D models are often less effective in 3D systems (20). The bioreactor system aims to recapitulate an environment similar to that of NETs *in vivo* by conserving the tumor’s stromal constituents, providing a 3D matrix for cellular organization, and incorporating constant nutrient flow. Therein, the model aims to serve as a more physiologically relevant tool for studying NETs than traditional culture methods and other 3D systems. Herein, we demonstrate the utility of this bioreactor system for evaluating the growth of NET surrogates generated from gastroenteropancreatic, thyroid, and bronchopulmonary NETs, validate multiple methods of monitoring tumor surrogate status, and provide methods for evaluating the efficacy of therapeutics on NET surrogates.

## 2 Materials and Methods

### 2.1 Cell Lines and Cell Culture

The study of gastroenteropancreatic, thyroid, and bronchopulmonary NETs suffers from a shared lack of cell line models upon which to conduct experiments. To demonstrate the utility of the bioreactor for culturing NETs of distinct tissue origin, cell lines representing each of these three types were used in this study. Furthermore, the lines used herein were selected for their public accessibility, widespread use as models in preclinical studies of NETs, and the robustness of their molecular characterization in the literature. Tumor constructs of medullary thyroid cancer (MTC) were generated using both wild-type (WT) and transgenic TT and MZ-CRC-1 cells. Transgenic MTC lines included TT and MZ-CRC-1 cells stably transfected with luciferase (TT-Luc, MZ-Luc), and TT cells stably transfected with red fluorescent protein (TT-RFP). GEP-NET constructs were generated using WT and transgenic BON-1 and QGP cells (human pancreatic NET cell lines). Transgenic BON-1 cells were stably transfected with luciferase (BON-Luc). Bronchopulmonary NET constructs were generated using WT UMC-11 and NCI-H727 lines as these lines are among the best characterized as typical carcinoid, the most common type of well-differentiated bronchopulmonary NET. WI-38 (human pulmonary fibroblast cell line), and HEK-293T (human embryonic kidney cells) were used as non-cancerous control lines. Transgenic BON-1, MZ-CRC-1, and TT cells were transfected with luciferase as previously described (26). Transgenic TT cells were likewise transfected with RFP. The BON-1 cell line was generously provided by Dr. Mark Hellmich (University of Texas, Galveston, TX). The QGP-1 cell line was obtained from Accegen (ABC-TC0918; Fairfield, NJ). Notably, despite the BON-1 line not being commercially available, BON-1 and QGP-1 cells are the most commonly used GEP-NET cell lines (27). All other cell lines were obtained from the American Type Culture Collection. The authenticity of all cell lines was validated with short tandem repeat profiling conducted by the UAB Heflin Genomics Core. All cell lines used were passaged under 15 times. BON-1, BON-1-Luc, MZ-Luc, and MZ-CRC-1 cells were cultured with Dulbecco’s Modified Eagle’s Medium/F12 (DMEM, Corning) supplemented with 10% Fetal Bovine Serum (FBS, Atlanta Biologicals) and 20 μg/ml Penicillin/Streptomycin (P/S, MP Biologics). TT, TT-Luc, and TT-RFP cells were cultured in Roswell Park Memorial Medium (RPMI 1640, Thermo Fisher) supplemented with 16% FBS and 1% P/S. N-Thyori, QGP, and UMC-11 cells were cultured in RPMI 1640 supplemented with 10% FBS and 20 μg/ml P/S. NCI-H727 cells were cultured in RPMI 1640 supplemented with 10% FBS, 20 μg/ml P/S, 1% Sodium Pyruvate (NaPyr, Thermo Fisher), 1% (4-(2-hydroxyethyl)-1-piperazineethanesulfonic acid buffer (HEPES, Thermo Fisher) and 1% glucose. WI-38 cells were cultured with Minimum Essential Medium- Eagle (Thermo Fisher) supplemented with 10% FBS, 1% NaPyr, and 1% Non-Essential Amino Acids (NEAA, Thermo Fisher). HEK-293T cells were cultured in DMEM supplemented with 10% FBS, 20 μg/ml P/S, and 1% L-Glutamate (Thermo Fisher).

### 2.2 PDMS Bioreactor Fabrication

PDMS bioreactors were molded using the Sylgard 184 Silicone Elastomer Kit (Dow) using a mold fabricated in-house by the UAB machine shop. The Sylgard 184 silicone elastomer and curing agent were mixed at a 10:1 ratio and this mixture was poured into the bioreactor mold. The mold was then baked for two hours at 80°C to cure the PDMS. PDMS bioreactors were removed from the mold and glass coverslips were attached to the top and bottom surfaces using freshly mixed PDMS to close the central chamber, as previously described (Goliwas et al., 2017). Once molded, each PDMS bioreactor measured 25 mm x 25 mm x 10 mm, with the cell/ECM chamber being 10 mm x 6 mm x 8 mm (**Figure 1**). PDMS bioreactors were autoclaved at 115°C for 15 minutes to sterilize before use.

### 2.3 Tissue-Engineered NET Model and Perfused Surrogate Preparation

To generate bioengineered NET models in the bioreactor, we used either patient-derived tumors (surrogates) or cell line derived mouse xenografts (constructs), respectively. All human subjects gave their informed consent for inclusion before they participated in the study. Human NETs were obtained after approval by the University of Alabama at Birmingham (UAB) Institutional Review Board (IRB) in accordance with all IRB/institutional regulations (IRB-021212003). A detailed description of patient demographic data, tumor site of origin, tumor grade, Ki-67, and success rate of patient tumor growth in the bioreactor is published elsewhere (28). Following resection, human specimens were placed in phosphate buffered saline (PBS) and transported on ice to the UAB Surgical Pathology Department for necessary clinical evaluations. Sterile tissue was then transported to the lab for processing. Xenografts were similarly handled. All animal procedures were conducted in accordance with UAB guidelines under Animal Project Number IACUC-20422. Upon receipt of a tumor specimen, approximately 250 mg of either human NETs or xenografts were then dissociated through a tissue dissociation sieve (Sigma Aldrich, 280 μm pores) and suspended in 70 μL of cell culture grade water. The extracellular matrix (ECM) was generated by adding 328.2 μL bovine type 1 collagen (BT1C, 9.6 mg/ml, Advanced Biomatrix), 45 μL 10X DMEM, 7.65 μL of sodium bicarbonate, and 450 μL Growth Factor Reduced Matrigel Basement Membrane (BM, Corning), comprising a 50/50 BT1C/Matrigel ECM as previously described (20). The mixture was homogenized by gentle pipetting, then injected *via* syringe into a PDMS bioreactor. During injection, the bioreactor is perforated by five 400 μm teflon coated wires located within an upstream wire guide affixed to the support bracket, to facilitate formation of the perfusion channels. Following ECM polymerization for 1 h, the wires were removed, generating five microchannels through the matrix. Bioreactors were then connected to a micro-peristaltic pump (Cole Parmer) and a media reservoir *via* Luer-fitted, peroxide-cured silicone tubing (Cole Parmer) with inflow/outflow stopcocks as depicted elsewhere (21). Surrogates/constructs were continuously perfused with 15 mL medium (Phenol Red Free DMEM/F12 supplemented with 10% FBS, Penicillin/Streptomycin) at a flow rate of 222 μl/min. Bioreactors were incubated at 37°C with supplementation of 5% CO_2_ for 3 to 30 days based on experimental endpoint. Culture media was changed every 3 days. Tubing and stopcocks were cleaned with 0.5% chlorhexidine and 70% EtOH before and after handling to prevent contamination. Further information on the preparation of the bioreactor are available in the **Supplemental Data**.

### 2.4 Non-Invasive Imaging

Following implantation, constructs containing murine TT-RFP xenografts (**Figures 4**, **7**) were imaged using an *In Vivo* Imaging System (IVIS) Lumina (Perkin-Elmer; Ex: 540, Em: 620). Regions of interest (ROI’s) were drawn around surrogates/constructs to measure luminescent or fluorescent signals. Fluorescence was measured as total radiant efficiency: a measure of photon flux from a source, normalized by exposure time, area of emission, and solid angle of the photon detector (sr), and defined as:


$$\text{Total Radiant Efficieny}=\left[\left(\text{photons}/\text{s}\right)/\left(\frac{\text{c}{\text{m}}^{2}}{\text{sr}}\right)\right]/\left(\frac{\mu \text{W}}{\text{c}{\text{m}}^{2}}\right)$$

Bioluminescence was measured as radiance: a measure of photon flux from a source, defined as:


$$\text{Radiance}=\left[\left(\text{photons}/\text{s}\right)/\left(\frac{\text{c}{\text{m}}^{2}}{\text{sr}}\right)\right]$$

IVIS images were analyzed using the Living Image Software Package (Perkin-Elmer). For luciferase-expressing cell line xenografts, constructs were incubated at 37°C with 150 μM D-Luciferin for 15 min prior to IVIS imaging. In experiments utilizing the NIR dye IR-783, 20 μM IR-783 was injected directly into the bioreactor for static incubation over 30 minutes (37°C, 5% CO2). After incubation, media was added to the reservoir system for perfusion over a 3-day washout period before imaging. Surrogates/constructs were imaged on day 3 after implantation (Ex: 780, Em: 845). Re-incubation with IR-783 occurred after each imaging session (or before every media change if surrogates/constructs were not being imaged) over the duration of bioreactor growth. After re-incubation, conditioned medium was perfused to flush channels for 1 hour, and media changed thereafter.

### 2.5 Treatment

Thailandepsin-A (TDP-A), a histone deacetylase inhibitor with known anti-NET activity (29) was dissolved in dimethylsulfoxide (DMSO). For evaluating the necessity of perfusion (**Figure 6**), TDP-A was administered at various concentrations into media, and culture wells filled to submerge the ECM. For monitoring xenograft response to treatment (**Figure 3**), TDP-A was injected into bioreactors upon media change, then perfused for the duration of the experiments depicted. In the same experiment, 6gy radiation was applied using an X-ray irradiator (Kimtron Inc.). In tumor constructs, the dose of TDP-A was increased 5x from the IC50 to account for the increased tissue volume and media perfusion (20, 29).

### 2.6 Histologic Analysis

**Figure 2:**
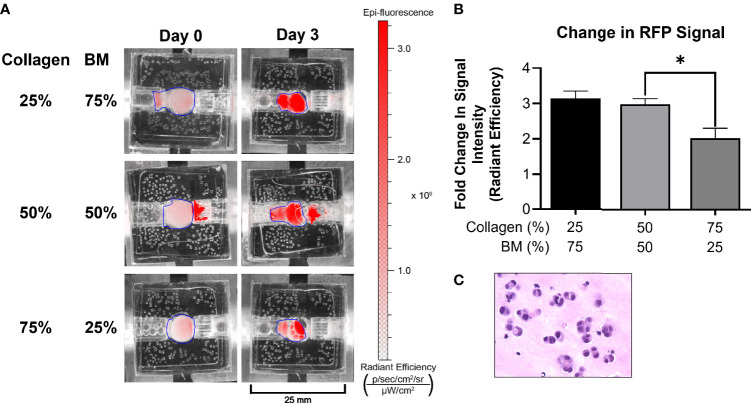
Optimization of ECM composition. **(A)** A medullary thyroid cancer (TT) cell line stably transfected with RFP grew optimally in 25/75 and 50/50 ECM composition, as depicted by IVIS imaging. All other experiments were performed utilizing the 50/50 ECM composition. **(B)** Following three days*’* culture, total radiant efficiency was comparable in membrane compositions of 25% BT1C/75% BM and 50% BT1C/50% BM. **(C)** H&E stained histologic section from 50/50 composition surrogate matrix following 3 days*’* culture (400x), depicting survival and epithelioid clustering of NET cells. "*" indicates significance at p < 0.05.

**Figure 3:**
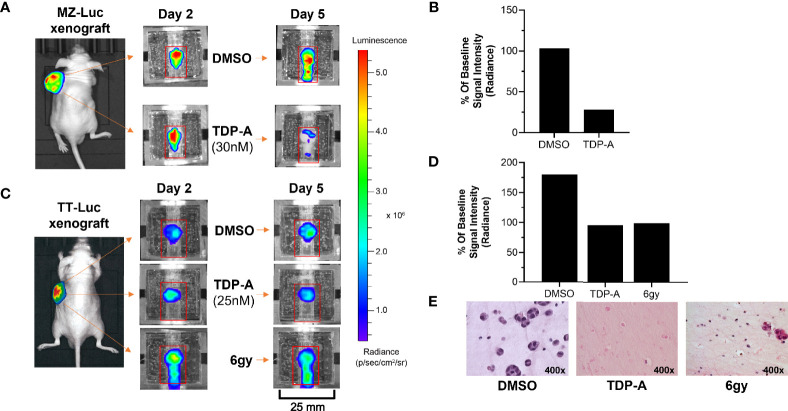
NET responses to anti-cancer therapeutics can be detected within the bioreactor. **(A, B)** MZ-Luc and **(C, D)** TT- Luc cells (5x10^6^ cells) were injected into nude mice and established for 2 weeks. Tumors were then implanted into the bioreactors for therapeutic testing. **(B)** A time-dependent decrease in bioluminescence signal of and MZ-Luc surrogate perfused with a 30nM dose of TDP-A showed a decrease from days 2 to 5 compared to DMSO control. **(D)** A similar result was detected in TT-Luc xenografts treated with either a 25 nM dose of TDP-A or a one-time radiation dose of 6 gy. **(E)** Sections of TT-Luc surrogates at day 5 depict decreased cell density and size in treated surrogates.

Bioreactors were fixed in 10% neutral buffered formalin for 24 h before tumor matrices were excised from the PDMS molds, placed into cassettes, and submerged in 70% ethanol for 24 h prior to paraffin embedding. Paraffin-embedded specimens were sectioned and stained with hematoxylin and eosin (H&E) before assessment by a pathologist for interpretation and quality assurance (**Figures 2**, **3**, **8**). For immunohistochemical staining (**Figures 8**, **9**), sections were re-hydrated using xylene and serial washes with decreasing concentrations of ethanol in deionized water. Slides were immersed in citrate buffer (pH 6, BioGenex) and placed into a pressure cooker for 10 min for antigen retrieval. Slides assessing the retention of NETs in the bioreactors were stained for Chromogranin A (CgA; Invitrogen Cat# MA5-14536) at a 1:100 dilution. Endothelial cells were identified using ETS-related gene (ERG; Abcam Cat # ab133264) at a 1:250 dilution. Lymphocytes were identified using an anti-CD45 antibody (Abcam Cat# ab10558) at a (1:100) dilution. Macrophages were identified using an anti-CD68 antibody (Abcam Cat#125212) at a 1:500 dilution. All sections were incubated with primary antibodies overnight at 4°C and an anti-rabbit biotinylated secondary antibody (Pierce goat anti-rabbit IgG, #31820) for one hour at room temperature. Slides were then stained with DAB chromogen (Dako Liquid DAB+ substrate K3468) and counterstained with hematoxylin.

### 2.7 Confocal Microscopy

**Figure 4:**
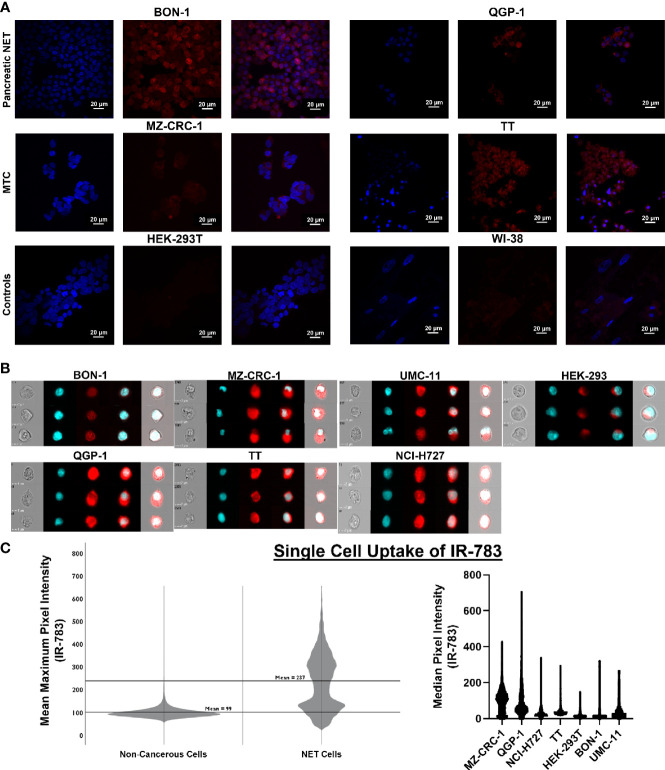
Single cell uptake of IR-783 is higher in NET cell lines than non-cancerous cell lines. **(A)** Confocal microscopy of human NET cell lines (BON-1, QGP-1, MZ-CRC-1, TT) and non-cancerous control cell lines (HEK-293T, WI-38) incubated with IR-783 (center) and stained with DAPI (left) demonstrated higher uptake of IR-783 in NET cells. IR-783 and DAPI stains are overlaid to depict localizaiton of IR-783 within the cells (right). **(B)** Human NET cell lines (BON-1, QGP-1, MZ-CRC-1, TT, UMC-11, NCI-H727) and non-cancerous controls cells (HEK-293T) were incubated with IR-783 and imaging flow cytometry was performed. **(C)** NET cell lines exhibited significantly higher median pixel intensities than non-cancerous cells (MZ=103.1, QGP-1 *=* 63.7, NCI-H727 *=* 50.6, BON-1 *=* 30.3, UMC-11 *=* 22.7, TT=73.6, and HEK-293T=11.9; p = <0.001). NET cells also exhibited higher mean maximum pixel intensity (237) than non-cancerous control cells (99) (p = <0.001).

NET (MZ-CRC-1, TT, BON-1, QGP-1) and non-cancerous cell lines (HEK-293T, WI-38) were plated on culture slides (Millipore) coated with fibronectin (Sigma Aldrich) and cultured for 48 h at 37°C. Cells were then incubated with 20 μM IR-783 for 30 min at 37°C, washed twice with PBS, and fixed with 10% formalin for 45 min at 4°C. Coverslips were mounted using Prolong-Gold w/DAPI (Invitrogen) and cured for 24 hours prior to imaging. Images were acquired using a Nikon A1 Confocal Laser Scanning Microscope (CLSM) with an Indocyanine Green filter cube and 633nm excitation laser (**Figure 4**).

### 2.8 Imaging Flow Cytometry (Imagestream)

NET (MZ-CRC-1, TT, NCI-H727, UMC-11, BON-1, QGP-1) and non-cancerous cell lines (HEK-293T) were incubated in 5 mL of 20 μM IR-783 & 10 μM Hoechst 33342 for 30 min at 37°C, then washed twice with PBS. They were then fixed with 10% formalin for 45 min at 4°C before resuspension in 50 μL PBS. Single cell images were then obtained with an Amnis Imagestream XMII (**Figure 4**). Fluorescent intensities were then analyzed using appropriate statistical analyses in SPSS Statistics v. 25 (IBM) and GraphPad Prism v. 8 (GraphPad Software).

### 2.9 Evaluating the Utility of IR-783

**Figure 5:**
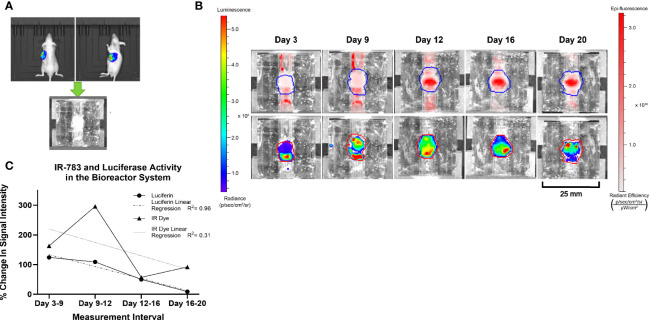
Similar trends in the intensity of Luciferase and IR-783 imaging measurements in BON-1 xenografts. **(A)** BON-LUC cells were subcutaneously injected into Nu-Nu mice, tumors grown, excised, and implanted into bioreactors. **(B)** Radiant signal obtained post luciferin and IR-783 administration was quantified *via* IVIS over the course of 20 days. **(C)** Goodness of fit to trendline was evaluated using simple linear regression (R^2^ *= *0.96 and 0.31 for Luciferase and IR-783*%* change respectively). A strong positive Spearman correlation (r_s_= 0.77) approached but did not achieve statistical significance (p= 0.051).

BON-1-Luc cells were injected subcutaneously into athymic Nu/Nu mice and a tumor grown over a period of 7 weeks. 100 μL of 90 mM D-Luciferin was injected intraperitoneally and imaging conducted *via* IVIS to determine the location of actively growing, viable cells. The growing portion was subsequently resected and implanted into the bioreactor system. 20 μM IR-783 was added to the medium (DMEM+Pen/Strep+FBS) initially and at each media change. Images were acquired post-incubation with IR-783, and after 15 min of incubation with 150 μM D-Luciferin at 3, 9, 12, 16, and 20 days of culture (**Figure 5**).

### 2.10 Propagation

After an initial growth period, bioreactors were prepared with 70% ethanol and placed onto a sterile field. The coverslip was pried from the scaffold and the tumor matrices sterilely excised from the bioreactors. Tumor matrices were passed through a tissue dissociation sieve as above (Section 2.3) and culture media added. Mixtures were then centrifuged at 1500rpm for 5 minutes. The pellet was mixed with a 50/50 mixture of BT1C and BM, 10x DMEM, sodium bicarbonate, and cell culture grade water before implantation into a new bioreactor as described above.

### 2.11 Statistical Analysis

**Figure 6:**
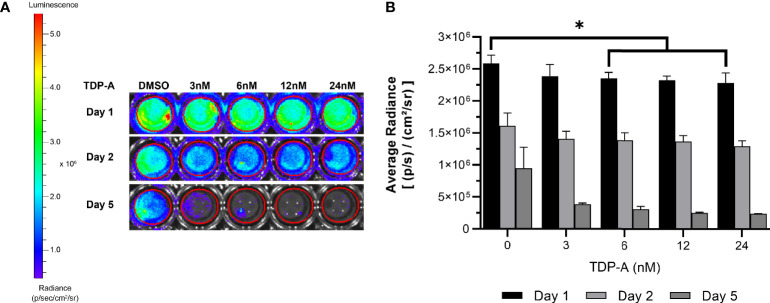
NET cells within ECM require perfusion for survival. **(A)** Cells from a TT-Luc xenograft were suspended in ECM and plated for culture without perfusion in triplicate. Cells were then treated with varying concentrations of TDP-A (0-24nM) or vehicle control (DMSO). Proliferation of TT-Luc cells over time was determined by luciferase activity. **(B)** Graphical display of a dose-dependent decrease in cell viability after TDP-A treatment. Wells treated with vehicle control had significantly higher radiance compared to all TDP-A doses over 3nM at all timepoints, but still exhibited a significant decrease in viability over the course of the experiment. “*” indicates significance at p < 0.05.

Continuous variables (i.e. total radiant efficiency, radiance) were evaluated for normality and homogeneity of variance between groups using Shapiro-Wilk and Levene’s tests. Two-way ANOVA and two-way repeated measures ANOVA were used to evaluate differences between groups/conditions where appropriate (**Figures 2**, **6**). Where normality was violated, the Kruskal-Wallis test was used (**Figure 4B**). Ordinary least squares linear regression and Spearman correlation was used to evaluate the relationship between fluorescent and bioluminescent intensities (**Figure 5**). All statistical analyses were performed in SPSS v. 26 and GraphPad Prism v. 8.

## 3 Results

### 3.1 Bioreactor Design and Optimization of ECM Composition for Surrogate Growth

The bioreactor system consists of a PDMS scaffold facilitating optically clear imaging through coverslips mounted on the top and bottom surfaces (**Figure 1**) and non-invasive monitoring of tumor cells. The central chamber of the scaffold houses the tumor surrogate matrix (**Figure 1A**), which is perforated by five microchannels through which media is perfused (**Figures 1B, C**). The scaffold is bracketed on either side for stabilization and connection to inflow and outflow stopcocks routing to a peristaltic pump (**Figure 1D**). The perfused matrix within the bioreactor is comprised of a growth factor reduced Matrigel Basement Membrane (BM) and bovine type 1 collagen (BT1C) mixture. BM was used primarily for its composition and physical properties. Its most abundant components, collagen IV and laminin, are the most abundant molecules in the ECM of the pancreatic islets (30, 31). BM also contributes stiffness to such hydrogel matrices, which is necessary to allow the ECM within the bioreactor to withstand the pressures of fluid flow (32). Additionally, Matrigel has been shown to facilitate proliferation and invasion of cancer cells in culture models (33). Growth factor reduced BM was chosen to minimize the dose of exogenous growth factors known to be present in Matrigel, which is significantly reduced compared to its standard formulation (31). However, to avoid possible confounding due to lot-to-lot variations, Matrigel lots were not intermixed within experiments. Type 1 collagen was chosen to augment the Matrigel ECM as it is highly secreted by pancreatic stellate cells, resident fibroblasts of both the endocrine and exocrine pancreas that are known to become cancer-associated fibroblasts and act as the primary drivers of fibrosis in pancreatic adenocarcinoma (34–36). Similarly, the desmoplastic reaction occurring in ~80% of medullary thyroid cancers is largely comprised of type 1 collagen, and the ECM of the lung is predominated by types I and II collagen (37–39). Additionally, the culture medium of BON-1 cells (pancreatic NET cell line) contained all 3 mammalian types of TGF‐β, and induces proliferation of mouse fibroblasts (40). As TGF-β is known to stimulate collagen synthesis (41), it is possible that it facilitates expansion of NET cells in their environment through stimulating production of key matrix elements such as type 1 collagen (42, 43). Based on this host of findings, type 1 collagen was chosen to provide further structure to the surrogate ECM. To empirically determine the ECM concentrations of BM and BT1C most conducive to surrogate growth, dose-response studies were conducted using TT cells (medullary thyroid cancer) stably transfected with red fluorescent protein (TT-RFP) (**Figures 2A, B**). After three days, total radiant efficiency was similar in compositions of 25% BT1C/75% BM and 50% BT1C/50% BM. Given the negligible difference in proliferation observed between these two configurations, ECM composition of 50% BT1C/50% BM were considered optimal and used in all other experiments. The presence of NET cells was verified histologically by sectioning and hematoxylin and eosin (H&E) staining of the fixed surrogate matrix composed of 50% BT1C/50% BM, depicting survival and epithelioid clustering of NET cells (**Figure 2C**).

### 3.2 Evaluating the Necessity of Perfusion

A key feature of the bioreactor model is the employment of a peristaltic pump to continuously perfuse nutrient medium through microchannels penetrating the surrogate volume, promoting cellular growth through nutrient recirculation and mechanical stimulation. The necessity of perfusion in culturing NETs within an ECM was evaluated using TT stably transfected with luciferase (TT-Luc) subcutaneously injected into athymic Nu/Nu mice. Tumors were harvested after three weeks and admixed within the earlier described optimal ECM during preparation in triplicate. These 3D cultures were then treated for 24 h with varied doses of thailandepsin A (TDP-A), a histone deacetylase inhibitor and potent NET growth inhibitor (29, 44). TDP-A doses ranged from 0-24 nM (IC_50_ = 6 nM in TT 2D cultures). Media was changed daily. Images were acquired at baseline, 2, and 5 days of culture (**Figures 6A, B**). While NET cell density as determined by bioluminescence expectedly decreased in treated cultures over time, similar decreases in bioluminescence were observed in the control cultures receiving DMSO alone, albeit to a lesser degree. The high rate of cell death within this culture environment despite absence of treatment supports that survival of NET cells within the ECM is greatly improved with media perfusion through the surrogate volume, as evidenced by the proliferation of TT-Luc control cells in **Figure 3**. These results align with previous evaluations of this model using a breast cancer/fibroblast co-culture (45).

### 3.3 Monitoring Proliferation and Therapeutic Efficacy in NET Constructs

**Figure 7:**
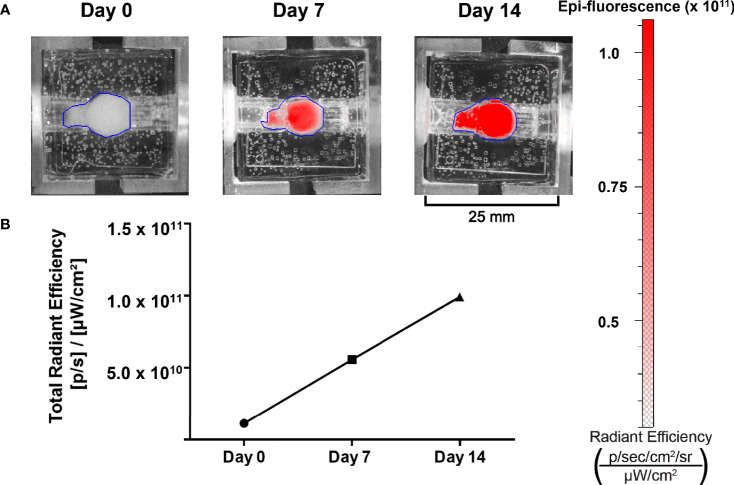
Growth of NET cell lines within the bioreactor system can be observed using spectral reporters. **(A)** IVIS imaging of surrogates containing TT-RFP cells at days 0, 7, and 14. **(B)** Region of interest measurement of fluorescent signals shows an increase between days 0 and 14.

To be a valid representation of NET phenotypes, surrogate imaging must be non-invasive and accurate. In accordance with this, the bioreactor’s design permits the use of imaging-based growth monitoring techniques. Hence, the capability to detect the proliferation of NET cells within the bioreactors was evaluated using a murine xenograft comprised of TT-RFP cells (**Figure 7A**). The tumor was implanted into a bioreactor and imaged over 14 days. A 10-fold linear increase in RFP signal was observed over the culture period, indicating surrogate growth in agreement with previous studies of the bioreactor system on breast cancer, lung cancer, and NETs (**Figure 7B**) (20, 21, 28, 45).

An important utility of the bioreactor system is evaluating the efficacy of therapeutics on NETs. This was demonstrated using MZ-CRC-1 (medullary thyroid cancer) and TT cells stably transfected with luciferase (MZ-Luc, TT-Luc) in murine xenografts generated as described above. Cell line xenografts were subjected to either a single dose of radiation (TT-Luc only), or 5x their respective IC_50_ of TDP-A in 2D culture. The IC_50_ was increased as such to account for the circulation of growth media, as previously described (20). MZ-Luc constructs were exposed to 30 nM of TDP-A or DMSO as control, and imaging was performed on days 2 and 5 (**Figure 3A**). Bioluminescence at endpoint was 103 and 28% of the baseline measurement (day 2) in the control and treated MZ-Luc constructs, respectively (**Figure 3B**). To verify these results, TT-Luc constructs were exposed to 25 nM TDP-A, a one-time radiation dose of 6gy, or DMSO, and images were acquired at days 2 and 5 (**Figure 3C**). Bioluminescence at endpoint was 180% of baseline measurement (day 2) in the control, compared to 86% in both the TDP-A and radiation treated TT-Luc constructs (**Figure 3D**). Following imaging, TT-Luc constructs were fixed and processed for histologic evaluation. H&E staining confirmed a decrease in cellularity in the treated constructs compared to controls **(** **Figure 3E** **)**.

### 3.4 Non-Invasive Growth Monitoring With IR-783

The prior experiments demonstrated that the bioreactor system can effectively allow growth of NET constructs for therapeutic response evaluations. However, while non-invasive fluorescent and bioluminescent imaging can be used to monitor tumor surrogates generated from cell lines, these methods cannot be employed to study patient-derived NETs; additionally, the introduction of such reporter genes has a myriad of limitations. First, this would require successful long-term culture of these neoplasms- a goal that is yet to be reproducibly achieved. Further, assuming successful culture and marker introduction, selection for marker expression would necessarily alter the composition and heterogeneity of the surrogate from the tumor *in situ*, conferring similar limitations to those faced with cell lines. An ideal reporter would be well-characterized, lack toxicity, and not rely on genomic insertion to be useful. Therefore, the utility of IR-783 was evaluated for its capability to indicate tumor status within the bioreactor system. Fluorescent dyes in the NIR excitability spectrum are promising imaging agents, as they exhibit minimal autofluorescence and display increased fluorescence once bound to intracellular macromolecules because of their rigidization (46). While traditional NIR dyes require chemical conjugation to target cancer cells specifically, IR-783 (Ex: 783 nm; Em: 845 nm) is an organic heptamethine cyanine NIR fluorescent dye internalized specifically into various types of cancer cells *via* increased expression of proteins from the organic anion transporter protein (OATP) family (47). As the affinity of IR-783 for NETs has not been illustrated, it was necessary to confirm that IR-783 would indeed be internalized avidly by NET cells. Additionally, the presence of normal stroma and inflammatory cells, known to comprise portions of tumor tissues, could potentially lead to disparities in fluorescent imaging results by interacting with IR-783 within the surrogate volume. Therefore, differences in the uptake of IR-783 between non-cancerous and NET cells were evaluated to determine the possible effects the presence of non-cancerous cells within the surrogate may have on fluorescent imaging studies. Single-cell internalization of the dye was first assessed in non-cancerous and NET cell lines with a CLSM. As reported previously (47), CLSM images with standardized fluorescent parameters exhibited a substantially higher degree of IR-783 retention in NET cells [TT, MZ-CRC-1, QGP-1(pancreatic NET line), and BON-1] as opposed to non-cancerous cells [HEK-293T (human embryonic kidney line) and WI-38 (fibroblast line); **Figure 4A**]. To quantify single cell uptake, NET cell lines and non-cancerous cells were analyzed *via* imaging flow cytometry (**Figure 4B**). The number of cells analyzed per cell line were: QGP-1 = 15,000; BON-1 = 6,000; TT=2,700; MZ-CRC-1 = 12,000; UMC-1 (pulmonary NET line) = 1,150; NCI-H727 (pulmonary NET line)=1,200; and HEK-293T=12,000. The median pixel intensity within IR-783 spectral emission wavelengths was 52 counts in NET cells and 30 in non-cancerous cells (p = <0.001). Individual cell line medians were the following: MZ=103.1, QGP=63.7, H727 = 50.6, BON=30.3, UMC=22.7, TT=13.6, and HEK=11.9 (p = <0.001). The mean maximum pixel intensity was 237 counts in NET cells and 99 in non-cancerous cells (p = <0.001; **Figure 4C**).

To draw meaningful conclusions on the trend of a tumor surrogate’s growth or the efficacy of therapeutic agents on the surrogate based on IR-783 uptake, the accuracy of those measurements for the changes in the number of NET cells *via* cell proliferation or death in culture needs evaluation. In addition, reliably tracking the changes in the number of viable NET cells within the bioreactor system using IR-783 requires that a correlation exists between cell number in the surrogates and the radiant intensity upon imaging. It has been demonstrated by other authors that bioluminescent imaging of cells stably transfected with the luciferase gene yields a strong quantitative correlation with cell number (48–50). Hence, a strong correlation between bioluminescent signal and the radiant efficiency measured upon imaging NET-Luc cells incubated with IR-783 would provide sufficient grounds for the utility of IR-783 to infer the changes in NET cell number. To evaluate this correlation, BON-1-Luc xenograft tumors were implanted into the bioreactor system to generate tumor constructs. The bioreactors were perfused with IR-783 as described above, and both fluorescent and bioluminescent images were acquired concurrently at days 3, 9, 12, 16, and 20 using an IVIS **(** **Figure 5** **)**. Because of the differing nature of the assays, raw comparisons of photon emission data were dissimilar. Therefore, data was normalized based on the degree of change from the previous measurements using each assay. Fit of trendlines was evaluated using simple linear regression (R^2^ = 0.96 and 0.31 for luciferase and IR-783 percent change, respectively). A strong positive Spearman correlation (r_s_ = 0.77) approached but did not achieve statistical significance (p = 0.051; **Figure 5C**).

### 3.5 Histologic Analysis of Patient-Derived Surrogates

#### 3.5.1 Retention of NET Cells

**Figure 8:**
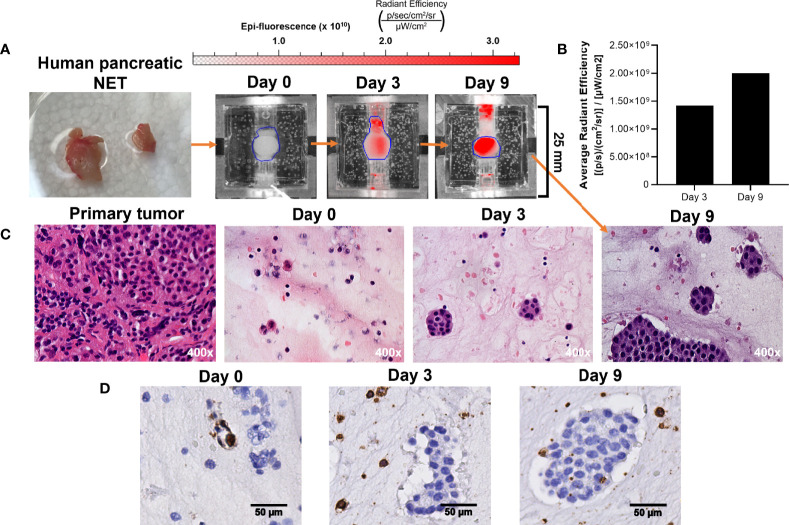
Growth of patient-derived NET surrogates is detectable using IR-783 and is consistent with increased density of NET cells histologically. **(A)** A human pancreatic NET was implanted into 3 separate bioreactors fixed at days 0, 3, and 9. **(B)** IVIS fluorescence imaging of the surrogate fixed at day 9 was performed on days 0, 3, and 9. Region of interest measurement of fluorescent signals depict a 41% increase in average radiant efficiency between days 3 and 9. **(C)** Photomicrographs of H&E stained histologic sections from the primary tumor and its surrogates following 0, 3 and 9 days in culture were acquired. Histological sections of surrogates depict epithelial clustering characteristic of NETs and increasing cell density from 0 to 9 days. **(D)** CD45 immunostaining of the bioreactors depicted in **(B)** identifies the retention of host immune cells within the proliferating tissue matrix.

While we have demonstrated that increased radiant efficiency upon imaging with IR-783 is supportive of NET in the bioreactor, this has not been confirmed using patient-derived NETs. Therefore, the proliferation of patient-derived NETs within the bioreactor was confirmed by acquiring histological sections of surrogate matrix derived from a primary human pancreatic NET (**Figure 8A**). Sections of the tumor were acquired before implantation, and equal volumes of tissue were implanted into three bioreactors. Surrogates were fixed at days 0, 3, and 9. IR-783 images of the surrogate fixed at day 9 depicted a 41% increase in average radiant efficiency from day 3 (**Figure 8B**). Fixed surrogate matrices were sectioned and stained with H&E, depicting increases in cell density during the culture period (**Figure 8C**). Interestingly, sections from these surrogates stained positively for CD45, depicting retention of host lymphocytes within the surrogate (**Figure 8D**). This indicates that immune interactions with NETs could also be studied with this model.

#### 3.5.2 Retention of Tumor Stroma

**Figure 9:**
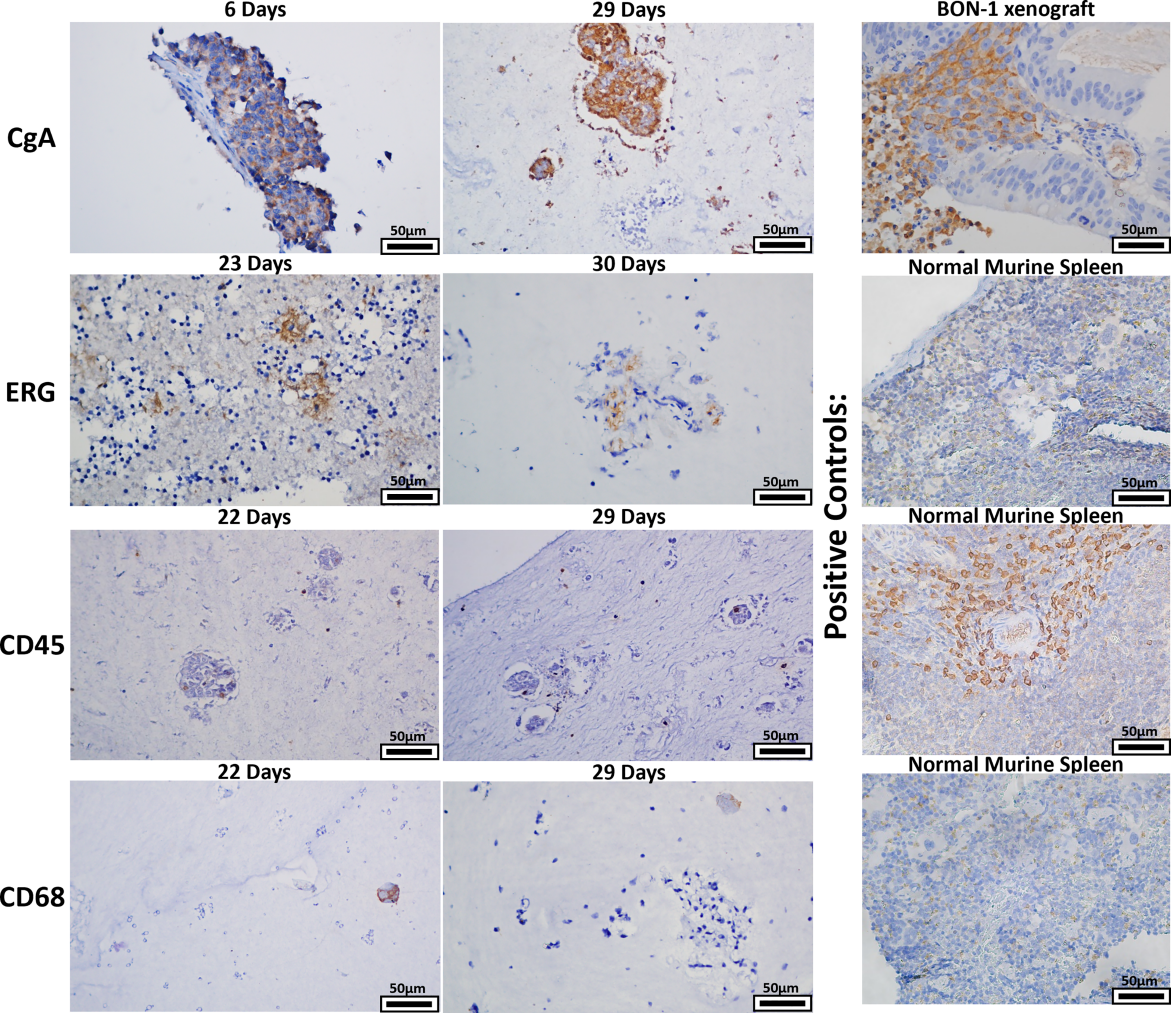
The bioreactor system retains key stromal populations over time. Six human NETs were implanted into multiple bioreactors. Each bioreactor was cultured to multiple endpoints based on experimental need or mechanical compromise. Sections of endpoint samples were stained with four different antibodies, depicting retention of NET and stromal cells over the culture period. Panels depicted are representative histological images of the surrogate matrices.

Given that a strength of the bioreactor is theoretical retention of stroma, retention of stromal populations was assessed using six resected human GEP-NETs. Two of these specimens were split into two separate bioreactors for this experiment, yielding eight bioreactor specimens total. Specimens were acquired and implanted per the methods and cultured to multiple endpoints based on experimental needs. Specimen endpoints ranged from 6-30 days. At endpoints, specimens were fixed, paraffin-embedded, and sectioned. Sections were stained with four antibodies targeting cell populations of interest (**Figure 9**). Sections from 8/8 bioreactors stained positively for CgA, ERG, and CD45. CD68 staining was positive in sections from 7/8 bioreactors, with the exception being a specimen with an endpoint of six days. While these results demonstrate the presence of endothelial cells, lymphocytes, and macrophages from the primary tumor interspersed with the tumor cells within the bioreactors, no discernable endothelial buds were noted.

### 3.6 Extended Growth and Propagation

**Figure 10:**
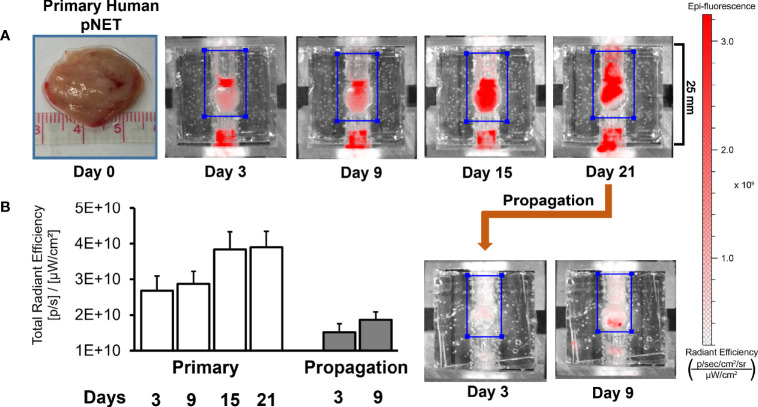
Human NET surrogates can be propagated in the bioreactor for long-term growth. **(A)** A human pNET was implanted into the bioreactor system and propagated successfully after 21 days of growth. The surrogate was terminated and fixed 9 days after propagation. **(B)** Quantitation of radiant efficiency data during the culture period.

While the bioreactor system allows for the culture of primary human NETs, a key goal of the model is to culture NETs long-term. As NETs are often slow-growing, extended observation periods are required to discern significant changes, particularly if studying the response of NETs to cytotoxic therapies (51). In this experiment, a patient-derived pancreatic NET was implanted into the bioreactor. To monitor growth during culture, 20 μM IR-783 was added to the medium. The tumor surrogate was grown for 21 days before cells were harvested from the matrix and transplanted into another bioreactor to propagate it (**Figure 10A**). Analysis of radiant efficiency depicted continued proliferation of the human NET surrogate after propagation for additional 9 days (**Figure 10B**). This suggests that patient-derived NET surrogates are viable through propagation, conferring that this system may allow for the culture of primary human NETs beyond the timeframe allowable by traditional cell culture methods (52, 53).

## 4 Discussion

In this study, we have determined that a composition of 50% BT1C and 50% BM facilitates growth of xenograft derived NETs in the bioreactor system to a greater degree than lower proportions of BM, and the same composition was used later to grow patient-derived NETs. We further determined that perfusion is necessary for NETs to proliferate within the ECM, and that proliferation of NET cells can be observed within the bioreactor system. Additionally, using NET xenografts, we demonstrated the ability to monitor a therapeutic response within the bioreactor system temporally. In order to evaluate the growth of patient-derived NETs within the bioreactor, we tested the difference in IR-783 uptake among NETs and non-cancerous cell lines, determining that uptake and retention of IR-783 is higher in NETs than non-cancerous cells. We then evaluated the trend and correlation between paired measurements using IR-783 and luciferase. While the trends between the two datasets were similar, and a strong positive correlation between them approached statistical significance, these data are insufficient to support a correlation between these two modalities. We then used IR-783 to monitor the growth of patient-derived pancreatic NET samples in the bioreactor, and histologically confirmed survival of NET cells, endothelia, lymphocytes, and macrophages within the bioreactor out to 29 days. Lastly, we demonstrated that patient-derived NETs can be propagated into subsequent bioreactors, indicating the potential for NETs to be cultured long-term within the bioreactor system.

Given the scarcity of methods to effectively culture and study primary NETs, the scope of the current literature on NETs is largely limited either to studies that can be performed immediately after resection or to experiments performed on cell lines. This narrow scope limits what can be learned about NET physiology and treatment. This obstacle is illustrated in the National Cancer Institute (NCI) goal to develop patient-derived xenografts (PDXs) as replacements for the NCI-60, a repository of 60 human cancer cell lines distributed for research (54). While PDXs are invaluable tools for studying tumor biology, attempts to establish them as models of NETs have been difficult (14, 55, 56). However, although few attempts have been made, PDX models in zebrafish have had a much higher success rate compared to attempts in mammalian organisms (57). The most notable study was performed by Gaudenzi et. al., wherein two GEP-NET metastases and four pituitary NETs were successfully xenografted into zebrafish embryos (58). Zebrafish models have a number of distinct advantages to the bioreactor system. Like murine xenografts, the organ systems, intact neuroendocrine system, and blood supply of zebrafish allow studies of metastasis and the systemic effects of neuroendocrine tumors. The rapid growth rate, transparent nature of zebrafish embryos, and availability of fluorescence-expressing zebrafish lines allow rapid and accessible study of NET migration *in vivo* with imaging techniques. Zebrafish PDX’s also have the benefit of requiring few NET cells (approximately 100 per embryo). However, they also come with disadvantages. Given that the optimal environmental temperature for zebrafish is 28**°**C, zebrafish PDX’s of NETs have been kept at 32**°**C as a compromise. While there may be short temperature fluctuations while handling the bioreactors for imaging, it is unknown what effects this lower temperature may have on NET metabolism and activity. Furthermore, tumors in zebrafish PDX’s have also been dissociated and sorted to enrich for NET cells in published studies, potentially losing key elements of the tumor stroma that are preserved in the bioreactor model. Lastly, the distinct microenvironment in zebrafish may affect patient derived NETs in ways that are not as well-characterized as those in mammalian model organisms.

Due to the difficulty of establishing PDXs as NET models, the necessity of cell lines becomes apparent. However, the relative dearth of NET cell lines diminishes the translational relevance of studies that employ them by decreasing the variety of specimens per disease that may be studied. Today’s array of NET cell lines includes approximately 23 that have been well-validated (59), compared to upwards of 200 used to study prostate cancer alone, for example (60). Even fewer of these are publicly available. Solving this problem by generating novel NET cell lines has also proven difficult. Many cell lines previously thought validated as NETs were discovered to either lack neuroendocrine characteristics (61, 62), have differentiated into radically different phenotypes, or have undergone senescence over the course of many passages (63). Furthermore, recent investigations into the genomic profiles of commonly used NET cell lines reveals atypical mutations, uncharacteristically rapid growth rates, and abnormal expression levels of cell surface markers common to NETs (63–65), indicating that these cell lines may not be entirely representative models of NET biology. By facilitating the effective culture of primary NET cells from patients, the bioreactor model could potentially mitigate a number of these issues.

Another method of modeling NETs is the use of organoids, which are three-dimensional cultures purposed to resemble a given organ in its morphology and characteristics. Organoids are often generated using stem cells from tissues of interest and a BM matrix, similar to the bioreactor model (56). Organoids have also been shown to remain genetically stable over years of passage and have been successfully used to study a multitude of cancers (66). Recently, methods to generate NET organoids have been successful using 2 cases of colorectal neuroendocrine carcinoma and 16 cases of pancreatic NET, portending successful models in the future (67, 68). However, these organoid models lack the tumor stroma, and of the organoid models of NETs that exist, most include a cell sorting step and consequently ignore this integral element of the tumor microenvironment. One such recent study established 25 lines of gastroenteropancreatic neuroendocrine neoplasms from patient samples, conducting a comprehensive genomic characterization of the organoids (69). However, all of the organoids successfully generated in this study were high-grade (Grade 3) NETs, or poorly differentiated neuroendocrine carcinomas, which represent a small minority of cases. Other promising techniques have recently been used to successfully generate organoids of well-differentiated small bowel NETs that likely include elements of the tumor stroma, which may prove useful as NET models in the future (70). Similar to organoids, the bioreactor permits extended growth of primary human tumor cells and provides a method by which relatively small tissue volumes can be utilized to establish multiple tumor surrogates. Multiple bioreactors also allow for the simultaneous analysis of different therapeutic interventions and phenotypic responses in cases where sufficient NET tissue can be obtained, such as in surgical debulking. Importantly, the bioreactor also retains key components of the tumor immune microenvironment, including lymphocytes and macrophages, which are known to influence the prognosis and progression of NETs (71). As such, this system may prove useful to model the behavior and interactions of these cells with NET cells in a dynamic tumor-like environment. Notably, the immune compartment within the bioreactor will likely shrink over time due to the absence of lymphatic germinal centers, and any T cells present in the bioreactor will likely develop an exhausted phenotype if they are not already exhausted upon implantation (72). Despite this, the bioreactor may prove useful in studying the acute response of a patient’s tumor to immunotherapies or the re-introduction of engineered lymphocytes (72).

While cells that express bioluminescent or fluorescent molecules are more easily and accurately monitored with imaging, dyes such as IR-783 that are retained in cancer cells are optimal for patient-derived tissues. However, a limitation of the studies herein are the properties of IR-783. As the dye is internalized *via* the organic anion transporter (OAT), the rationale for the specificity of high IR-783 retention in cancer cells is based on studies highlighting differences in the type and number of OATs expressed between cancerous and non-cancerous cells (73–76). While the preferential uptake of IR-783 in cancer cells has been reliably observed (47), IR-783 is internalized in tissue types highly expressing OATs, including the renal proximal tubule, hepatic parenchyma, and choroid plexus (77, 78). Notably, our studies show IR-783 uptake in the non-cancerous HEK-293T and WI-38 lines. Unpublished data have also revealed IR-783 retention within pancreatic islets. However, as illustrated in **Figure 4**, the degree of uptake was vastly lower in these non-cancerous cells. Additionally, this work also highlights that inherent differences exist between the bioluminescent signal elicited from luciferase-expressing cells, and the fluorescent signal observed in those same cells after incubation with IR-783. This difference exists understandably, as fluorescence using IR-783 is dependent upon the activity and abundance of OAT proteins in cancer cells, while the activity of luciferase depends upon the presence of oxygen, magnesium, and ATP (79).

A key limitation of this work is the low number of biological replicates. This was partly due to the rarity of feasibly resectable human NETs; but was ultimately due to the volume of tissue used to generate each surrogate. While 250 mg of tissue was used per bioreactor, experiments were not performed to determine a minimum tissue volume necessary for generating a viable tumor surrogate in this model. Rather, smaller volumes of tissue had been used in previous versions of the bioreactor. These models suffered from a number of deficiencies, one of them being the propensity for the perfusion channels to collapse. Collapse of these channels often occurred in succession, with the resultant increase in pressure pulling away the tissue’s adhesions to the inner chamber and causing washout of the specimen through the media outflow. Increasing the surface area of the matrix chamber, and hence the amount of tissue necessary, was found to decrease the incidence of channel collapse and preserved of the structural integrity of the specimen. Related to this, it is also necessary to carefully monitor the bioreactor for mechanical compromise or media leakage, particularly for the time period immediately following activation of the flow-perfusion pump. This, along with careful sterile technique and routine cleaning of the tubing and external bioreactor components with 0.5% chlorhexidine solution and 70% ethanol prevented contamination. Furthermore, the use of GFR Matrigel in the tumor matrix within the bioreactor is a limitation. GFR Matrigel undergoes specific iterative exclusion for various growth factors, followed by ammonium sulfate precipitation, size-exclusion chromatography, and SDS-PAGE. As a result, it contains a relatively low abundance of growth factors compared to the unmodified variety (31). This process enriches the formulation for matrix structural proteins, but nonetheless retains a level of growth factors and other signaling molecules that may influence the cellular behavior (growth, therapeutic response, immunogenicity) within the bioreactor. Matrigel does afford a relatively high level of stiffness that facilitated the perfusion of the tumor matrix in this model. Furthermore, while optimization of conditions is necessary for any 3D culture matrix, optimizing alternatives to Matrigel such as synthetic scaffolds can be costly and time-consuming. Additionally, methods for the appropriate arrangement or cross-linking of synthetic scaffolds often requires specialized equipment or chemical alterations that mandate cells be inoculated into a pre-formed matrix, rather than homogenously mixed (80, 81). This trade-off may be particularly impactful in the case of slow-growing cell populations such as those of neuroendocrine cancers. These tradeoffs are largely responsible for Matrigel’s continued popularity in cancer research particularly, although advances in synthetic scaffolds will likely replace Matrigel in the future (81, 82). Among the trade-offs of using Matrigel is the nature of its biological derivation. As it is harvested from the murine Englebreth-Holm-Swarm sarcoma cells, there is the possibility of lot-to-lot variations in its composition that has been demonstrated in some studies (81, 83). While we attempted to mitigate the influence this may have on our work by not intermixing lots, this variation is an obstacle to precise control of the environmental conditions within the bioreactor’s tumor matrix.

Herein, we have demonstrated that the bioreactor system may be effectively used for an array of applications in NETs. The persistence of a heterogeneous NET and stromal population within the system provides a unique advantage over other culture models that may improve the translational relevance of preclinical studies. Given the ability to propagate NETs within the bioreactor, it may also be useful as a tool for long-term growth of well-differentiated NETs, which has proven challenging in the past. Further characterization of NETs grown over long durations within the system will be essential for understanding the breadth of applications the bioreactor and similar systems are suited for.

## Data Availability Statement

The original contributions presented in the study are included in the article/**Supplementary Material**. Further inquiries can be directed to the corresponding author.

## Ethics Statement

The studies involving human participants were reviewed and approved by The UAB Institutional Review Board. The patients/participants provided their written informed consent to participate in this study. The animal study was reviewed and approved by The UAB Institutional Animal Care and Use Committee.

## Author Contributions

Conceptualization and methodology, SJ, BH, ZA, JW, JBe, AF, KG, HC, JR and RJ-S. Formal analysis, SJ, BH, KG, AF. Investigation, SJ, BH, JW, KG, HC, JR, RJ-S. Resources, ZA, JBe, JBi, HC, JR, RJ-S. Writing—original draft preparation, BH, KG, RJ-S. Writing—review and editing, all authors. Supervision, administration, and funding acquisition, HC, JR, RJ-S. All authors have read and agreed to the published version of the manuscript.

## Funding

Research support includes the North American Neuroendocrine Tumor Society Basic/Translational Science Investigator Grant, the Central Surgical Association Enrichment Award, the UAB Center for Clinical and Translational Sciences Training Grant (TL1 TR001418), the National Cancer Institute of the National Institutes of Health under award numbers K08CA234209 and R21CA226491-01A1, and the UAB Medical Scientist Training Program (T32 GM008361). Research reported in this publication was also supported by the NCI Cancer Center Support Grant (P30 CA013148), the UAB Comprehensive Flow Cytometry Core (NIH P30 AR048311 & P30 AI27667), and the UAB High Resolution Imaging Facility.

## Conflict of Interest

The authors declare that the research was conducted in the absence of any commercial or financial relationships that could be construed as a potential conflict of interest.

## Publisher’s Note

All claims expressed in this article are solely those of the authors and do not necessarily represent those of their affiliated organizations, or those of the publisher, the editors and the reviewers. Any product that may be evaluated in this article, or claim that may be made by its manufacturer, is not guaranteed or endorsed by the publisher.

## References

[1] Das S Dasari A. Epidemiology, Incidence, and Prevalence of Neuroendocrine Neoplasms: Are There Global Differences? Curr Oncol Rep (2021) 23:43. doi: 10.1007/s11912-021-01029-7 33719003 PMC8118193

[2] Dasari A Shen C Halperin D Zhao B Zhou S Xu Y. Trends in the Incidence, Prevalence, and Survival Outcomes in Patients With Neuroendocrine Tumors in the United States. JAMA Oncol (2017) 3:1335–42. doi: 10.1001/jamaoncol.2017.0589 PMC5824320 28448665

[3] Taal BG Visser O. Epidemiology of Neuroendocrine Tumours. Neuroendocrinology (2004) 80(suppl 1):3–7. doi: 10.1159/000080731 15477707

[4] Hofland J Kaltsas G de Herder WW. Advances in the Diagnosis and Management of Well-Differentiated Neuroendocrine Neoplasms. Endocr Rev (2020) 41:371–403. doi: 10.1210/endrev/bnz004 PMC7080342 31555796

[5] Singh S Granberg D Wolin E Warner R Sissons M Kolarova T. Patient-Reported Burden of a Neuroendocrine Tumor (NET) Diagnosis: Results From the First Global Survey of Patients With Nets. J Global Oncol (2016) 3:43–53. doi: 10.1200/JGO.2015.002980 PMC5493232 28717741

[6] Farley HA Pommier RF. Treatment of Neuroendocrine Liver Metastases. Surg Oncol Clin N Am (2016) 25:217–25. doi: 10.1016/j.soc.2015.08.010 26610783

[7] Oronsky B Ma PC Morgensztern D Carter CA Nothing But NET. A Review of Neuroendocrine Tumors and Carcinomas. Neoplasia (2017) 19:991–1002. doi: 10.1016/j.neo.2017.09.002 29091800 PMC5678742

[8] Pavel M O''Toole D Costa F Capdevila J Gross D Kianmanesh R. ENETS Consensus Guidelines Update for the Management of Distant Metastatic Disease of Intestinal, Pancreatic, Bronchial Neuroendocrine Neoplasms (NEN) and NEN of Unknown Primary Site. Neuroendocrinology (2016) 103:172–85. doi: 10.1159/000443167 26731013

[9] Lee L Ito T Jensen RT. Everolimus in the Treatment of Neuroendocrine Tumors: Efficacy, Side-Effects, Resistance, and Factors Affecting its Place in the Treatment Sequence. Expert Opin Pharmacother (2018) 19:909–28. doi: 10.1080/14656566.2018.1476492 PMC6064188 29757017

[10] Pavel ME Hainsworth JD Baudin E Peeters M Hörsch D Winkler RE. Everolimus Plus Octreotide Long-Acting Repeatable for the Treatment of Advanced Neuroendocrine Tumours Associated With Carcinoid Syndrome (RADIANT-2): A Randomised, Placebo-Controlled, Phase 3 Study. Lancet (2011) 378:2005–12. doi: 10.1016/S0140-6736(11)61742-X 22119496

[11] Foster DS Jensen R Norton JA. Management of Liver Neuroendocrine Tumors in 2018. JAMA Oncol (2018) 4:1605–6. doi: 10.1001/jamaoncol.2018.3035 30178021

[12] Wiedmann MW Mössner J. Safety and Efficacy of Sunitinib in Patients With Unresectable Pancreatic Neuroendocrine Tumors. Clin Med Insights Oncol (2012) 6:381–93. doi: 10.4137/CMO.S7350 PMC3511053 23226079

[13] Kaderli RM Spanjol M Kollár A Bütikofer L Gloy V Dumont RA. Therapeutic Options for Neuroendocrine Tumors: A Systematic Review and Network Meta-Analysis. JAMA Oncol (2019) 5:480. doi: 10.1001/jamaoncol.2018.6720 30763436 PMC6459123

[14] Yang Z Zhang L Serra S Law C Wei A Stockley TL. Establishment and Characterization of a Human Neuroendocrine Tumor Xenograft. Endocr Pathol (2016) 27:97–103. doi: 10.1007/s12022-016-9429-4 27067082

[15] Gahete MD Jiménez-Vacas JM Alors-Pérez E Herrero-Aguayo V Fuentes-Fayos AC Pedraza-Arévalo S. Mouse Models of Endocrine Tumors. J Endocrinol (2019) 240:R73–96. doi: 10.1530/JOE-18-0571 30475226

[16] Baretti M Zhu Q Zahurak M Bhaijee F Xu H Engle EL. Prognostic Implications of the Immune Tumor Microenvironment in Patients With Pancreatic and Gastrointestinal Neuroendocrine Tumors. Pancreas (2021) 50:719–26. doi: 10.1097/MPA.0000000000001831 34016898

[17] Takkenkamp TJ Jalving M Hoogwater FJH Walenkamp AME. The Immune Tumour Microenvironment of Neuroendocrine Tumours and its Implications for Immune Checkpoint Inhibitors. Endocrine-Related Cancer (2020) 27:R329–43. doi: 10.1530/ERC-20-0113 32590336

[18] Bussard KM Mutkus L Stumpf K Gomez-Manzano C Marini FC. Tumor-Associated Stromal Cells as Key Contributors to the Tumor Microenvironment. Breast Cancer Res (2016) 18:84. doi: 10.1186/s13058-016-0740-2 27515302 PMC4982339

[19] Mafficini A Scarpa A. Genetics and Epigenetics of Gastroenteropancreatic Neuroendocrine Neoplasms. Endocr Rev (2019) 40:506–36. doi: 10.1210/er.2018-00160 PMC6534496 30657883

[20] Goliwas KF Richter JR Pruitt HC Araysi LM Anderson NR Samant RS. Methods to Evaluate Cell Growth, Viability, and Response to Treatment in a Tissue Engineered Breast Cancer Model. Sci Rep (2017) 7:14167. doi: 10.1038/s41598-017-14326-8 29074857 PMC5658356

[21] Goliwas KF Ashraf HM Wood AM Wang Y Hough KP Bodduluri S. Extracellular Vesicle Mediated Tumor-Stromal Crosstalk Within an Engineered Lung Cancer Model. Front Oncol (2021) 11:1404. doi: 10.3389/fonc.2021.654922 PMC8103208 33968758

[22] Bryce NS Zhang JZ Whan RM Yamamoto N Hambley TW. Accumulation of an Anthraquinone and its Platinum Complexes in Cancer Cell Spheroids: The Effect of Charge on Drug Distribution in Solid Tumour Models. Chem Commun (2009) (19):2673–5. doi: 10.1039/b902415h 19532917

[23] Trédan O Galmarini CM Patel K Tannock IF. Drug Resistance and the Solid Tumor Microenvironment. JNCI: J Natl Cancer Inst (2007) 99:1441–54. doi: 10.1093/jnci/djm135 17895480

[24] Pickup MW Mouw JK Weaver VM. The Extracellular Matrix Modulates the Hallmarks of Cancer. EMBO Rep (2014) 15:1243. doi: 10.15252/embr.201439246 25381661 PMC4264927

[25] Hongisto V Jernström S Fey V Mpindi J-P Kleivi Sahlberg K Kallioniemi O. High-Throughput 3D Screening Reveals Differences in Drug Sensitivities Between Culture Models of JIMT1 Breast Cancer Cells. PLoS One (2013) 8:e77232. doi: 10.1371/journal.pone.0077232 24194875 PMC3806867

[26] Wyche TP Dammalapati A Cho H Harrison AD Kwon GS Chen H. Thiocoraline Activates the Notch Pathway in Carcinoids and Reduces Tumor Progression *In Vivo* . Cancer Gene Ther (2014) 21:518–25. doi: 10.1038/cgt.2014.57 PMC4270822 25412645

[27] Ney A Canciani G Hsuan JJ Pereira SP. Modelling Pancreatic Neuroendocrine Cancer: From Bench Side to Clinic. Cancers (Basel) (2020) 12:3170. doi: 10.3390/cancers12113170 PMC7693644 33126717

[28] Herring B Whitt J Aweda T Ou J Guenter R Lapi S. A Growth Model of Neuroendocrine Tumor Surrogates and the Efficacy of a Novel Somatostatin-Receptor-Guided Antibody-Drug Conjugate: Perspectives on Clinical Response? Surgery (2020) 167:197–203. doi: 10.1016/j.surg.2019.04.073 31543319 PMC8162105

[29] Jang S Janssen A Aburjania Z Robers MB Harrison A Dammalapati A. Histone Deacetylase Inhibitor Thailandepsin-a Activates Notch Signaling and Suppresses Neuroendocrine Cancer Cell Growth *In Vivo* . Oncotarget (2017) 8:70828–40. doi: 10.18632/oncotarget.19993 PMC5642598 29050323

[30] Llacua LA Faas MM de Vos P. Extracellular Matrix Molecules and Their Potential Contribution to the Function of Transplanted Pancreatic Islets. Diabetologia (2018) 61:1261–72. doi: 10.1007/s00125-017-4524-8 PMC6449002 29306997

[31] Hughes CS Postovit LM Lajoie GA. Matrigel: A Complex Protein Mixture Required for Optimal Growth of Cell Culture. Proteomics (2010) 10:1886–90. doi: 10.1002/pmic.200900758 20162561

[32] Anguiano M Morales X Castilla C Pena AR Ederra C Martínez M. The Use of Mixed Collagen-Matrigel Matrices of Increasing Complexity Recapitulates the Biphasic Role of Cell Adhesion in Cancer Cell Migration: ECM Sensing, Remodeling and Forces at the Leading Edge of Cancer Invasion. PLoS One (2020) 15:e0220019. doi: 10.1371/journal.pone.0220019 31945053 PMC6964905

[33] Ruud KF Hiscox WC Yu I Chen RK Li W. Distinct Phenotypes of Cancer Cells on Tissue Matrix Gel. Breast Cancer Res (2020) 22:82. doi: 10.1186/s13058-020-01321-7 32736579 PMC7395363

[34] Apte M Pirola R Wilson J. Pancreatic Stellate Cells: A Starring Role in Normal and Diseased Pancreas. Front Physiol (2012) 3:344. doi: 10.3389/fphys.2012.00344 22973234 PMC3428781

[35] Bynigeri RR Jakkampudi A Jangala R Subramanyam C Sasikala M Rao GV. Pancreatic Stellate Cell: Pandora's Box for Pancreatic Disease Biology. World J Gastroenterol (2017) 23:382–405. doi: 10.3748/wjg.v23.i3.382 28210075 PMC5291844

[36] Wu Y Zhang C Jiang K Werner J Bazhin AV D’Haese JG. The Role of Stellate Cells in Pancreatic Ductal Adenocarcinoma: Targeting Perspectives. Front Oncol (2021) 10:3044. doi: 10.3389/fonc.2020.621937 PMC7841014 33520728

[37] Parker AL Cox TR. The Role of the ECM in Lung Cancer Dormancy and Outgrowth. Front Oncol (2020) 10:1766. doi: 10.3389/fonc.2020.01766 33014869 PMC7516130

[38] Koperek O Scheuba C Cherenko M Neuhold N De Micco C Schmid KW. Desmoplasia in Medullary Thyroid Carcinoma: A Reliable Indicator of Metastatic Potential. Histopathology (2008) 52:623–30. doi: 10.1111/j.1365-2559.2008.03002.x 18370959

[39] Koperek O Scheuba C Puri C Birner P Haslinger C Rettig W. Molecular Characterization of the Desmoplastic Tumor Stroma in Medullary Thyroid Carcinoma. Int J Oncol (2007) 31:59–67. doi: 10.3892/ijo.31.1.59 17549405

[40] Beauchamp RD Coffey RJ Lyons RM Perkett EA Townsend CM Moses HL. Human Carcinoid Cell Production of Paracrine Growth Factors That can Stimulate Fibroblast and Endothelial Cell Growth. Cancer Res (1991) 51:5253. 1913648

[41] Ignotz RA Massagué J. Transforming Growth Factor-Beta Stimulates the Expression of Fibronectin and Collagen and Their Incorporation Into the Extracellular Matrix. J Biol Chem (1986) 261:4337–45. doi: 10.1016/S0021-9258(17)35666-1 3456347

[42] Chaudhry A Oberg K Gobl A Heldin CH Funa K. Expression of Transforming Growth Factors Beta 1, Beta 2, Beta 3 in Neuroendocrine Tumors of the Digestive System. Anticancer Res (1994) 14:2085–91. doi: 10.3109/02841869309083898 7840504

[43] Modlin IM Shapiro MD Kidd M. Carcinoid Tumors and Fibrosis: An Association With No Explanation. Am J Of Gastroenterol (2004) 99:2466. doi: 10.1111/j.1572-0241.2004.40507.x 15571597

[44] Jaskula-Sztul R Xu W Chen G Harrison A Dammalapati A Nair R. Thailandepsin a-Loaded and Octreotide-Functionalized Unimolecular Micelles for Targeted Neuroendocrine Cancer Therapy. Biomaterials (2016) 91:1–10. doi: 10.1016/j.biomaterials.2016.03.010 26994874 PMC4865460

[45] Marshall LE Goliwas KF Miller LM Penman AD Frost AR Berry JL. Flow-Perfusion Bioreactor System for Engineered Breast Cancer Surrogates to be Used in Preclinical Testing. J Tissue Eng Regener Med (2017) 11:1242–50. doi: 10.1002/term.2026 PMC5985445 25950420

[46] Harrison VSR Carney CE MacRenaris KW Waters EA Meade TJ. Multimeric Near IR–MR Contrast Agent for Multimodal *In Vivo* Imaging. J Am Chem Soc (2015) 137:9108–16. doi: 10.1021/jacs.5b04509 PMC4512902 26083313

[47] Yang X Shi C Tong R Qian W Zhau HE Wang R. Near Infrared Heptamethine Cyanine Dye-Mediated Cancer Imaging. Clin Cancer Res (2010) 16:2833–44. doi: 10.1158/1078-0432.CCR-10-0059 PMC2871283 20410058

[48] Close DM Xu T Sayler GS Ripp S. *In Vivo* Bioluminescent Imaging (BLI): Noninvasive Visualization and Interrogation of Biological Processes in Living Animals. Sensors (Basel Switzerland) (2011) 11:180–206. doi: 10.3390/s110100180 PMC3274065 22346573

[49] Konopka R Hýzdalová M Kubala L Pacherník J. New Luminescence-Based Approach to Measurement of Luciferase Gene Expression Reporter Activity and Adenosine Triphosphate-Based Determination of Cell Viability. Folia Biol (Praha) (2010) 56:66–71. 20492758 10.14712/fb2010056020066

[50] Shi Y Liu W Zheng H Li Z Shi X Cai S. Imaging of Pre-Mrna Splicing in Living Subjects Using a Genetically Encoded Luciferase Reporter. BioMed Opt Express (2018) 9:518–28. doi: 10.1364/BOE.9.000518 PMC5854055 29552390

[51] Joo WD Visintin I Mor G. Targeted Cancer Therapy–are the Days of Systemic Chemotherapy Numbered? Maturitas (2013) 76:308–14. doi: 10.1016/j.maturitas.2013.09.008 PMC4610026 24128673

[52] Arhlund L Nilsson O Kling-Petersen T Wigander A Theodorsson E Dahlstroum A. Serotonin-Producing Carcinoid Tumour Cells in Long-Term Culture Studies on Serotonin Release and Morphological Features. Acta Oncol (1989) 28:341–6. doi: 10.3109/02841868909111204 2663047

[53] Lundqvist M Ouberg K. *In Vitro* Culture of Neuroendocrine Tumors of the Pancreas and Gut. Acta Oncol (1989) 28:335–9. doi: 10.3109/02841868909111203 2472821

[54] Ledford H. Us Cancer Institute Overhauls Cell Lines. Nature (2016) 530:391. doi: 10.1038/nature.2016.19364 26911756

[55] Krampitz GW George BM Willingham SB Volkmer J-P Weiskopf K Jahchan N. Identification of Tumorigenic Cells and Therapeutic Targets in Pancreatic Neuroendocrine Tumors. Proc Natl Acad Sci (2016) 113:4464. doi: 10.1073/pnas.1600007113 27035983 PMC4843455

[56] Kawasaki K Fujii M Sato T. Gastroenteropancreatic Neuroendocrine Neoplasms: Genes, Therapies and Models. Dis Models Mech (2018) 11(2):dmm029595. doi: 10.1242/dmm.029595 PMC5894937 29590641

[57] Germano G Silvia C Alessandra D Maria Celeste C Luca P Giovanni V. Fishing for Neuroendocrine Tumors. Endocrine-Related Cancer (2020) 27:R163–76. doi: 10.1530/ERC-19-0437 32252025

[58] Gaudenzi G Albertelli M Dicitore A Würth R Gatto F Barbieri F. Patient-Derived Xenograft in Zebrafish Embryos: A New Platform for Translational Research in Neuroendocrine Tumors. Endocrine (2017) 57:214–9. doi: 10.1007/s12020-016-1048-9 27481363

[59] Grozinsky-Glasberg S Shimon I Rubinfeld H. The Role of Cell Lines in the Study of Neuroendocrine Tumors. Neuroendocrinology (2012) 96:173–87. doi: 10.1159/000338793 22538498

[60] Sobel RE Sadar MD. Cell Lines Used in Prostate Cancer Research: A Compendium of Old and New Lines—Part 2. J Urol (2005) 173:360–72. doi: 10.1097/01.ju.0000149989.01263.dc 15643173

[61] Van Buren G Rashid A Yang AD Abdalla EK Gray MJ Liu W. The Development and Characterization of a Human Midgut Carcinoid Cell Line. Clin Cancer Res (2007) 13:4704. doi: 10.1158/1078-0432.CCR-06-2723 17699847

[62] Ellis LM Samuel S Sceusi E. Varying Opinions on the Authenticity of a Human Midgut Carcinoid Cell Line – Letter. Clin Cancer Res (2010) 16:5365. doi: 10.1158/1078-0432.CCR-10-2550 20959409

[63] Boora GK Kanwar R Kulkarni AA Pleticha J Ames M Schroth G. Exome-Level Comparison of Primary Well-Differentiated Neuroendocrine Tumors and Their Cell Lines. Cancer Genet (2015) 208:374–81. doi: 10.1016/j.cancergen.2015.04.002 26087898

[64] Hofving T Arvidsson Y Almobarak B Inge L Pfragner R Persson M. The Neuroendocrine Phenotype, Genomic Profile and Therapeutic Sensitivity of GEPNET Cell Lines. Endocrine-Related Cancer (2018) 25:367–80. doi: 10.1530/ERC-17-0445 PMC5827037 29444910

[65] Vandamme T Beyens M Peeters M Van Camp G de Beeck KO. Next Generation Exome Sequencing of Pancreatic Neuroendocrine Tumor Cell Lines BON-1 and QGP-1 Reveals Different Lineages. Cancer Genet (2015) 208:523. doi: 10.1016/j.cancergen.2015.07.003 26341699

[66] Blokzijl F de Ligt J Jager M Sasselli V Roerink S Sasaki N. Tissue-Specific Mutation Accumulation in Human Adult Stem Cells During Life. Nature (2016) 538:260–4. doi: 10.1038/nature19768 PMC5536223 27698416

[67] Fujii M Shimokawa M Date S Takano A Matano M Nanki K. A Colorectal Tumor Organoid Library Demonstrates Progressive Loss of Niche Factor Requirements During Tumorigenesis. Cell Stem Cell (2016) 18:827–38. doi: 10.1016/j.stem.2016.04.003 27212702

[68] April-Monn SL Wiedmer T Skowronska M Maire R Schiavo Lena M Trippel M. Three-Dimensional Primary Cell Culture: A Novel Preclinical Model for Pancreatic Neuroendocrine Tumors. Neuroendocrinology (2020) 111:273–87. doi: 10.1159/000507669 32241015

[69] Kawasaki K Toshimitsu K Matano M Fujita M Fujii M Togasaki K. An Organoid Biobank of Neuroendocrine Neoplasms Enables Genotype-Phenotype Mapping. Cell (2020) 183:1420–35.e21. doi: 10.1016/j.cell.2020.10.023 33159857

[70] Au - Ear PH Au - Li G Au - Wu M Au - Abusada E Au - Bellizzi AM Au - Howe JR. Establishment and Characterization of Small Bowel Neuroendocrine Tumor Spheroids. JoVE (2019) (152):e60303. doi: 10.3791/60303 PMC7654522 31657801

[71] Cives M Pelle’ E Quaresmini D Rizzo FM Tucci M Silvestris F. The Tumor Microenvironment in Neuroendocrine Tumors: Biology and Therapeutic Implications. Neuroendocrinology (2019) 109:83–99. doi: 10.1159/000497355 30699437

[72] Boucherit N Gorvel L Olive D. 3D Tumor Models and Their Use for the Testing of Immunotherapies. Front Immunol (2020) 11. doi: 10.3389/fimmu.2020.603640 PMC7758240 33362787

[73] Ballestero MR Monte MJ Briz O Jimenez F Martin FG-S Marin JJG. Expression of Transporters Potentially Involved in the Targeting of Cytostatic Bile Acid Derivatives to Colon Cancer and Polyps. Biochem Pharmacol (2006) 72:729–38. doi: 10.1016/j.bcp.2006.06.007 16844096

[74] Mikkaichi T Suzuki T Tanemoto M Ito S Abe T. The Organic Anion Transporter (Oatp) Family. Drug Metab Pharmacokinet (2004) 19:171–9. doi: 10.2133/dmpk.19.171 15499184

[75] Marzolini C Tirona RG Kim RB. Pharmacogenomics of the OATP and OAT Families. Pharmacogenomics (2004) 5:273–82. doi: 10.1517/phgs.5.3.273.29831 15102542

[76] Sarakbi WA Mokbel R Salhab M Jiang WG Reed MJ Mokbel K. The Role of STS and OATP-B Mrna Expression in Predicting the Clinical Outcome in Human Breast Cancer. Anticancer Res (2006) 26:4985–90. 17214375

[77] Wu JB Shi C Chu GC-Y Xu Q Zhang Y Li Q. Near-Infrared Fluorescence Heptamethine Carbocyanine Dyes Mediate Imaging and Targeted Drug Delivery for Human Brain Tumor. Biomaterials (2015) 67:1–10. doi: 10.1016/j.biomaterials.2015.07.028 26197410 PMC4736505

[78] Nigam SK Bush KT Martovetsky G Ahn S-Y Liu HC Richard E. The Organic Anion Transporter (Oat) Family: A Systems Biology Perspective. Physiol Rev (2015) 95:83–123. doi: 10.1152/physrev.00025.2013 25540139 PMC4281586

[79] de Wet JR Wood KV DeLuca M Helinski DR Subramani S. Firefly Luciferase Gene: Structure and Expression in Mammalian Cells. Mol Cell Biol (1987) 7:725–37. doi: 10.1128/mcb.7.2.725-737.1987 PMC365129 3821727

[80] Redmond J McCarthy H Buchanan P Levingstone TJ Dunne NJ. Advances in Biofabrication Techniques for Collagen-Based 3D *In Vitro* Culture Models for Breast Cancer Research. Mater Sci Eng: C (2021) 122:111944. doi: 10.1016/j.msec.2021.111944 33641930

[81] Aisenbrey EA Murphy WL. Synthetic Alternatives to Matrigel. Nat Rev Mater (2020) 5:539–51. doi: 10.1038/s41578-020-0199-8 PMC7500703 32953138

[82] Franchi-Mendes T Eduardo R Domenici G Brito C. 3D Cancer Models: Depicting Cellular Crosstalk Within the Tumour Microenvironment. Cancers (Basel) (2021) 13. doi: 10.3390/cancers13184610 PMC8468887 34572836

[83] Talbot NC Caperna TJ. Proteome Array Identification of Bioactive Soluble Proteins/Peptides in Matrigel: Relevance to Stem Cell Responses. Cytotechnology (2015) 67:873–83. doi: 10.1007/s10616-014-9727-y PMC4545444 24744128

