# Supplementary material for: Ex Vivo Modeling of Human Neuroendocrine Tumors in Tissue Surrogates
Source: Front Endocrinol (Lausanne). 2021 Dec 23;12:710009. doi: 10.3389/fendo.2021.710009 (PMC8734644; doi:10.3389/fendo.2021.710009)
Supplement: Supplementary file 1 [file DataSheet_1.docx]

**Supplemental Data S1. Bioreactor Components and Preparation**

*Components/setup needed for 1 bioreactor*

-3 long pieces 1/16” ID tubing **(Cole Parmer, EW-06411-62)**

-3 short pieces 1/16” ID tubing **(Cole Parmer, EW-06411-62)**

-2 straight tubing connectors (1/16” OD) **(McMaster-Carr, 5047K71)**

-3 female Luer fittings (1/16” OD) **(McMaster-Carr, 51525K319)**

With Luer sides covered in Teflon tape **(Fisher, 22-163741)**

-4 male Luer fittings (1/16” OD) **(McMaster-Carr, 51525K326)**

-4 male caps **(McMaster-Carr, 51525K334)**

-2 female caps **(McMaster-Carr, 51525K333)**

-2 pieces of Teflon tape **(Fischer, 22-163741)**

- PDMS bioreactor with coverslips attached **(Refer to Methods/UAB Machine Shop)**

- Rubber syringe plug with two metal canals, both with male Luer fittings attached via 1/16” tubing (1 long, 1 short; **part #** **above**)

*Sterilization of bioreactor components:*


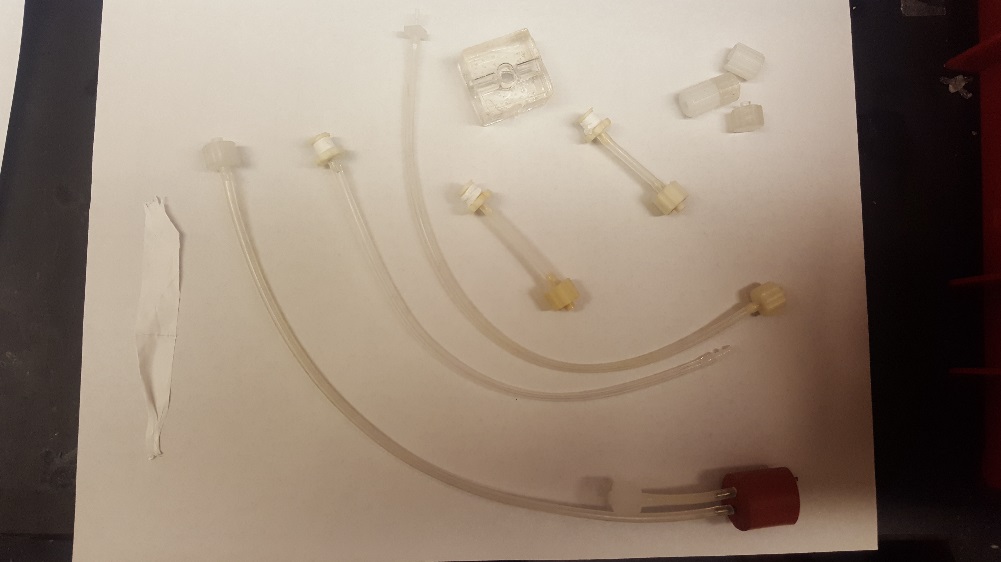


Luer Straight tubing connectors (blue circles)

**Supplemental Figure S1**. **Autoclavable Bioreactor Components**. These components are autoclaved for sterilization at 115°C for 15 min with a 15 min drying time. Assemble as many of the components together before autoclaving.


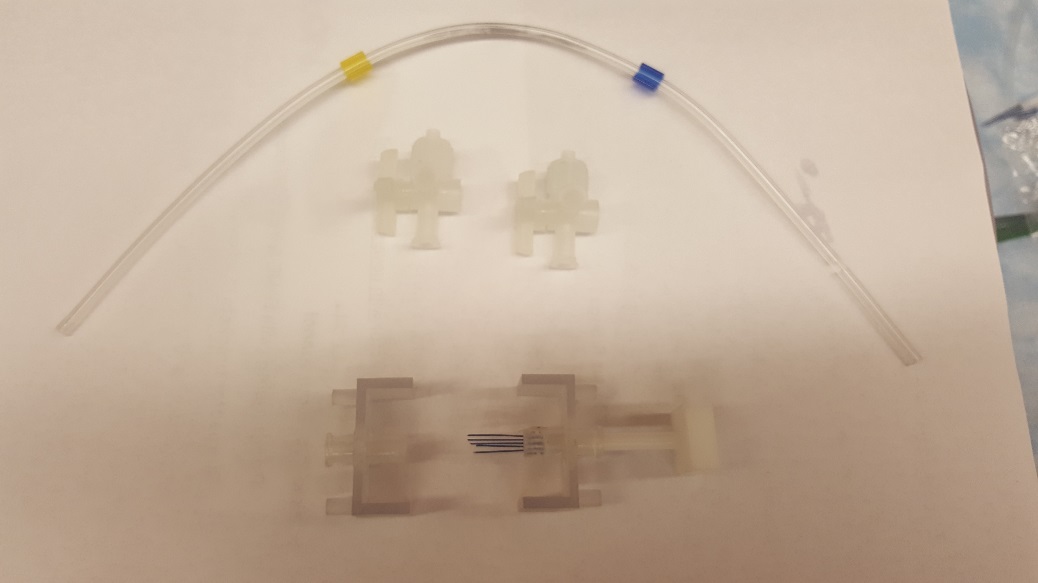


**Supplemental Figure S2**. **Non-Autoclavable Bioreactor Components**. These components should be soaked/washed under gentle agitation on an orbital shaker in the following solutions: a 10% bleach solution followed by 5x 5 min washes in sterile DI H20, a 0.5% chlorhexidine solution for 30 min, followed by 5x 5 min washes in sterile DI H_2_0, followed by a single soak in 70% ethanol or isopropanol for 30 min. This is followed by UV irradiation in a fume hood for 30 min and storage in a sterile container or immediate use.

Non-Autoclavable parts include:

-Pump tubing (with BLUE & YELLOW brackets) **(Cole Parmer, EW-74906-36)**

-Bioreactor brackets (1 inflow with wire guide & 1 outflow) **(UAB Machine Shop)**

-2 stopcocks (3 way Luer) **(Cole Parmer, EW-74906-04)**

**No preparation/sterilization needed:**

- Sterile Syringe Filter **(Fischer, 09-754-29)****attached atop media reservoir, for adding new media*

- Sylgard 184 **(Electron Microscopy Sciences, 24236-10)**

- Media reservoir syringe **(Fischer, 22-124-967)**

- BD Luer-lock syringe 1ml **(Fischer, 14-823-30)**

- Gauze sponges **(Fischer, 22-415-469)**

- 18G needles **(Fischer, 14-826-SD)**

*PREPARATION (DAY BEFORE)*

1. Clean & autoclave OR soak, agitate, and irradiate all components for bioreactor experiment.

2. Thaw **GFR Matrigel** overnight (stored in -20^o^C) by leaving in the refrigerator (-4^o^C) on ice.

3. Assemble **PDMS bioreactor housing as below.**

**
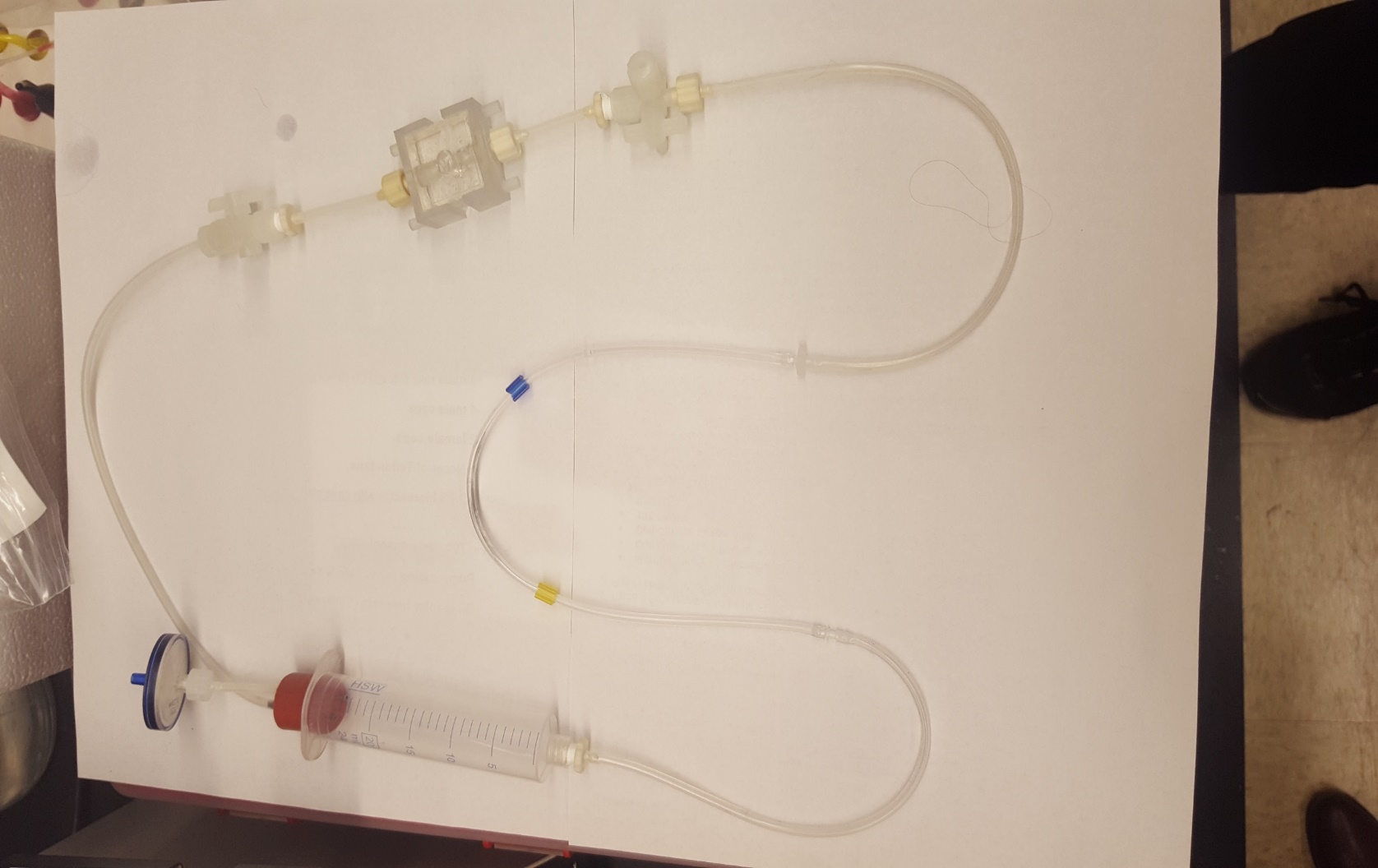
**

**Supplemental Figure S3**. **Full Bioreactor Assemblage**. All sterilized components, shown connected without ECM injected.

**BIOREACTOR SET UP.** *All operations are performed in a cell culture hood, on a sterile drape and using sterile technique*.

1. Warm the following items in the water bath:

- **Cell culture media**

2. Ready collagen/Matrigel gel components:

- GFR Matrigel (thawed in ice; **Corning, 354230**)
- Collagen (Type I Bovine, ~9.6 mg/ml) **(Advanced BioMatrix, #133-A)**
- Cell Culture Grade Water **(Fischer, BP2819-1)**
- 10X DMEM **(Sigma, D2429)**
- NaOH **(Sigma, S8761)**

3. Pass cells through tissue dissociation sieve, suspend cells in 70 uL cell culture-grade H20.

Need ~0.525e^6^ cells per 100 uL ECM

Prep 900 uL ECM per bioreactor

4.725e^6^ cells per bioreactor

**Approximately 4,725,000/ (cells/ml) = are the ideal cell suspension needed per bioreactor**

4. Add the following components in order into a **1.5 ml microcentrifuge tube.** This amount is enough for 1 bioreactor.

**50%** **9.6 mg/ml collagen (bovine high conc.) + 50% (v/v) Matrigel (900uL ECM w/ cells)**

450 µl Matrigel GFR -- use normal pipet

45 µl 10X DMEM -- use normal pipet

7.65 µl Sodium Bicarbonate (drop-wise) -- use normal pipet

328.2 µl Collagen (drop-wise) (9.6 mg/ml bovine)* -- use ^#^***viscous pipet***

70 µl Cell culture water containing cells -- use normal pipet

**Volume will change if stock collagen concentration is not 9.6 mg/ml*

#*Viscous pipet* ***(*Microman pipet 1000ul, Gilson,** **F148506)**

5. Gently mix using 18G needle **(Fischer, 14-826-5G)** & 1mL syringe **(Fischer, 14-823-30)** until homogeneous.

Sodium bicarbonate begins the solidification process, so if setting up multiple bioreactors, put in sodium bicarbonate right before mixing and injecting.

6. Inject ECM into bioreactor from the outflow side, with inflow bracket and Luer fittings/stopcock attached.

Working with bioreactor, be careful not to break the coverslip. Insert outflow brackets with attached Luer fittings and capped stopcock after injection.

7. Incubate samples for 1 hour at 37° C in a sterile container to allow for ECM polymerization.

8. After 1hrs, confirm that the matrix solidified, and wrap female Luer fittings with Teflon tape to prevent leaking.

9. Attach stopcocks to 2 short assembled tubings with Luer pieces.

10. Prepare media reservoir with 20mL syringe **(Fischer, 22-124-967).**

11. Prime upstream (inflow) stop cock. Note: *If imaging, attach stopcocks and cap Luer ends.*

Use 1mL Luer-lock syringe to push media through one of the short tubing pieces attached to a stopcock.

12. Gently remove wire guide from bioreactor fittings, and wrap inflow side with teflon tape.

13. Attach short assembled pieces + stock cock onto BOTH ends of the stopcock.

Primed short piece goes on the inflow side.

11. Using the 20mL Luer-lock syringe, gently push 15mL media into reservoir through inflow side.

Stop cock setting should have media flow into the reservoir, NOT towards the bioreactor.

12. Connect the rest of the exterior tubing and media reservoir to each side of the bioreactor.

13. Tighten all connections thoroughly to prevent media leakage.

13. Move bioreactors to incubator and, insert tubing into peristaltic pump, and turn ON the pump.

Ensure that all stopcocks are open for flow and that the direction of flow is correct.
 Tubing color: yellow-blue, **Volumetric flow rate: (4.2 rpm, 1 dyne / cm^2^; equates to ~222 μl/min).**

14. Incubate at 37 for duration of experiment, changing media every 3 days.

Media is gently aspirated from the upstream (inflow) stopcock, and added via the sterile filter atop the media reservoir.
